# “All the Difference in the World”: The Nature of Difference and Different Natures

**DOI:** 10.1177/0048393120917947

**Published:** 2020-05-27

**Authors:** Paolo Heywood

**Affiliations:** 1 University of Cambridge, Cambridge, UK

**Keywords:** anthropology, ontology, multinaturalism, perspectivism, essentialism

## Abstract

This article begins by examining the status of “difference” in representations of perspectivist cosmologies, which are themselves often represented as radically different to Euro-American cosmologies. The established reading of perspectivism emphasizes this radical difference by focusing upon the objects of difference in perspectivism (bodies, for example, rather than souls). This article experiments instead with reading perspectivism as radically resembling Euro-American thought in its conceptualization of the nature of difference, that is, the form that difference takes as a relation. It argues that in schematic representations of Amerindian and Euro-American cosmologies, difference for both is always a matter of institution and construction, and resemblance is a matter of essence and necessity. Thus, paradoxically, arguments about radical difference may in fact be read to assert an underlying essentialism as to the nature of difference itself. I conclude by proposing that we abandon conceptions of the nature of difference, in favor of a focus on “styles” of difference, and discuss some non-anthropological examples of this approach, as well as instances of different “styles” of difference from my own fieldwork.

## 1. Introduction

“The essence of the comparative method is to make sense of differences, not collapse them” (Strathern 1987, 286). “Making sense of differences” could well stand as a shorthand for the essence, not just of the comparative method, but of anthropology as a discipline. But if “differences” are essential to the nature of anthropology, what, if anything, is essential to difference? Is there a necessary “conception of relationship” (Strathern 2011, 97), a “nature,” to this necessary anthropological object?

Such questions have arisen in a number of forms over the past few decades, perhaps in particular as a result of the recognition that “making sense of differences” is far from being a peculiarly anthropological enterprise, or, if it is, that anthropology, at least in this sense, is not a peculiar activity. Transforming that enterprise into an object of ethnographic knowledge, into one of those very “differences” we aim to make sense of, makes it much harder to imagine that any one “conception” of difference as a relation (“cultural” difference, say, though that may mean different things, as I suggest below) should take precedence over another. Different people make sense of differences in different ways.

Perhaps the most well-known recent instance of this recognition is a set of arguments which have stemmed in part from ethnographic insights into perspectivist notions of difference. These arguments have promised a revolution in the way in which we think about difference: from epistemological to ontological, from cultural to natural, from the kinds of difference with which anthropologists normally concern themselves to “radical alterity.” I will not rehearse these arguments here because my intention is not to intervene in the debates which have resulted, but to return—briefly, and as a starting point for further discussion—to some of those original ethnographic insights. In other words, I am less interested, for present purposes, in where those debates have gone than in where they began, and in this beginning as a paradigmatic instance of a different “conception” of difference as a relation. I will attempt to show that while from one perspective—that usually adopted toward them in anthropological literature—they do of course effect some radical alterations to our thinking about difference, from another—the perspective I adopt here—they in fact reinforce perfectly traditional anthropological conceptions of what kind of difference is at work. I will then try to use this suggestion as a platform from which to advance an alternative methodological proposal, namely that we take different “styles” of doing difference seriously, and abandon claims about the “nature” or “essence” of difference still implicit in our most recently different conceptions of it. I will do so by drawing on two of my own ethnographic cases, as well as work in cognate disciplines.

## 2. Different Differences

“‘Nature’ itself is pluralised” (Skafish 2014). That is the startling consensus that has emerged from ethnographic studies of perspectivism over the past two decades. Worlds, not just worldviews, may differ; material bodies, not ephemeral souls, may be the sources of such difference. Here I also proceed from the premise that nature is multiple, but take a different perspective on the meaning of that premise.

I take the notion that “nature is multiple” to mean not that “there are multiple worlds,” but simply to describe the fact that anthropologists (along with a great many others) use the word “nature” to refer to multiple different things. Among these things, two, in particular, are of interest for the argument here: first, an object, or perhaps even objects in general (world, matter, the environment, animals, volcanoes, jaguars, and so on); second, a property of objects (the essential and constitutive aspect of something that makes it that thing and not another, and without which it becomes something else).

The argument I will make here is that at least some of the complexity and power of arguments concerning the multiplicity of nature is derived from the fact that as well as describing radical difference and equivocation, often they themselves equivocate over these two possible referents of the word.

My invocations of “perspectivism” and its cosmological opposite, which I will here call “naturalism,”^1^ are not ethnographic, but anthropological, in that I am interested in how they have appeared in anthropological arguments, rather than their relationship to objects and behaviors in the world: in their status as indexing a “relation of intelligibility between two cultures,” not as “veridical reflections” of those cultures (Viveiros de Castro 2014, 190), and in the broader arguments that have emerged from this relation. I will briefly sketch out some of perspectivism’s relevant features below, largely as they emerge from the work of perhaps its most well-known exponent, Eduardo Viveiros de Castro (1996, 1998, 2004a, 2004b, 2007, 2011a, 2012, 2014). This is not intended as a library re-study of the classic form, for which I have neither the space nor the expertise. My purpose instead is to clarify some of the implications of this work for anthropology: the meta-problems involved in describing and accounting for its apparent radical difference from our own understandings of difference. The problem, as I have suggested above, is not simply how to describe a particular ethnographic difference, but how to describe a difference over difference itself.

In the schema of perspectivist arguments, “we” understand difference to be a matter of culture or worldview. For “us,” human beings are naturally identical and members of the same species, but spiritually and mentally diverse. Our bodies are the same, but our souls and our minds are different. We also live in the same world, but our ideas about it vary. There is “a physical continuity and a metaphysical discontinuity between humans and animals” (Viveiros de Castro 1998, 479, 2004a, 475). “Spirit or mind . . . raises us above animals . . . [and] makes each person unique before his or her fellow beings” (Viveiros de Castro 1998, 479, 2004a, 475). In that sense, we exist in natural unity and cultural diversity. This corresponds to the first sense in which I used the word “nature” above: human bodies are the same sorts of object; the world is one thing. Cultures are multiple. Nature is not.

In the ethnographic literature on Amazonia, on the other hand, the situation is the mirror inverse of “ours.” There, “humanity” is “the universal form of the subject” (Viveiros de Castro 1998, 470, 2004a, 468), and “the common point of reference for all beings of nature is not humans as a species but rather humanity as a condition” (Descola 1986, 120; Viveiros de Castro 1998, 472). Hence, Amerindian words which designate “human being” mark “a social condition of personhood,” not “a natural species” (Viveiros de Castro 1998, 476). Hence, equally, the proliferation of origin myths in which the primordial condition is not a natural animality from which human beings must distinguish themselves through culture, but a humanity which animals proceed to lose (Viveiros de Castro 1998, 471, 2004a, 464, 2014, 68-69). All humans, which, as a designator of personhood not species includes some animals, share the same spirit, soul, or perspective upon the world (“jaguars see blood as manioc beer, vultures see the maggots in rotting meat as grilled fish”; Viveiros de Castro 1998, 470). The body, in contrast, is an “envelope” or “clothing” which conceals an inner human essence (Viveiros de Castro 1998, 471), but is also responsible for the different objects seen (Viveiros de Castro 1998, 478, 2004a, 474). Here, in other words, we have a metaphysical continuity of subjecthood and a physical discontinuity of objects. Souls, which mark the person as human, are the same, while bodies, bundles of “affects and capacities” which “differentiate perspectives,” are different (Viveiros de Castro 1998, 478, 2004a, 474, 2004b, 6; see also Harris and Robb 2012). The worlds thus perceived also differ (hence, famously, “multinaturalism”) even as the form of the human perspective does not. Cultures are not multiple. Nature is.

The dilemma to which this gives rise is the status of that description itself: is *that* difference, between natures as different and cultures as different, a cultural one? Claiming that it is would diminish the radical nature of the difference in question (over nature itself) as well as leading inevitably to the conclusion that Amerindians are wrong, not only in the content of their worldview but in believing it not to be one. Culturalism, when applied to perspectivism “implies the negation or delegitimization of its object” (Viveiros de Castro 2004b, 5, 2014, 87). Anyone who has attempted to teach the topic to undergraduates may not have to imagine the disheartening look on a student’s face, when, having got to grips with the ethnographic accounts themselves, they declare “so in their culture difference is natural,” and the realization of the paradoxical implications of that statement set in. The option of describing it as a natural difference in seemingly recursive fashion is as hard to swallow as the culturalist option, as the practice of designating some peoples as “naturally” different from others has a more than troubled political history (for discussion of which in relation to multinaturalism see, for example, Vigh and Sausdal 2014). This troubled political history is the source of some critiques of anthropology’s “ontological turn” that see it as exoticising and othering of its subjects. As Vigh and Sausdal (2014) put it,

Though ontology, as it glides from defining things, concepts and ideas to denoting people, groups and entire civilizations, is not necessarily articulated as rooted and territorialized, it is nonetheless theoretically constructed as naturalised and essentialised, internally coherent and bounded, as incommen-surable worlds. (2014, 65)

These are what I mean by the “meta-problems” to which the by now already classic plethora of ethnographic descriptions of perspectivism in Amazonia (and elsewhere; see, for example, Pedersen et al. 2007; Willerslev 2007) have given rise. They are fundamental problems in anthropology, given the foundational status of notions surrounding “cultural difference” in the discipline, and help to explain why these ethnographic descriptions have given rise to a whole corpus of methodological and epistemological reflections on the status of anthropological knowledge itself (e.g., Henare et al. 2007; Holbraad 2012; Viveiros de Castro 2004b, 2011a, 2014). They have been bombs placed under Western philosophy, as Latour (2009) describes them. Among these, of note is the work of Eduardo Viveiros de Castro, who has, over several decades, authored significant contributions to both the ethnographic literature on perspectivism in Amazonia (indeed, systematizing it for the first time) and the methodological consequences of this work for the broader discipline.

Although he has elsewhere referred to his characterization of Amerindian cosmologies as “tactical (procedural) quintessentialism” (Viveiros de Castro 2011b, 165), in perhaps his most sustained reflections on the methodological implications of Amerindian perspectivism for the basic anthropological projects of translation and comparison, he refers to the indigenous version of these as “equivocation.” He goes on to elevate it to the “condition of possibility” of the anthropological enterprise (Viveiros de Castro 2004b, 10, 2014, 89). Equivocation is about dealing with problems of the above form: not just misunderstandings but, to paraphrase Roy Wagner’s oft-cited aphorism, the fact that our misunderstandings of them may not be the same as their misunderstandings of us (Wagner 1981, 20). Equivocation is neither direct culturalist translation of the traditional anthropological kind nor essentialization or objectification, but “the relational positivity of difference” (Viveiros de Castro 2004b, 12, 2014, 74). It is not about “discovering the common referent . . . to two different representations” (Viveiros de Castro 2004b, 6) but instead about not “losing sight of the difference concealed within equivocal ‘homonyms’” (Viveiros de Castro 2004b, 7). The question I wish to pose here, however, is what kind of difference? Critiquing, in the same piece, work which imposes cultural constructionist frameworks onto indigenous perspectivist cosmologies, Viveiros de Castro (2004b, 16) notes, following Wagner (1981, 51), that ‘there is all the difference in the world” between the two (see also Viveiros de Castro 2014, 62-63), and that reducing one to the frameworks of the other is “to imagine an overly simple form of relation between them” (Viveiros de Castro 2004b, 16). But that, surely, is precisely the question, in many ways the most basic of anthropological questions: are they so different? Or, instead, what is it in our descriptions of them that causes such differences to appear, and can these descriptions be differentiated yet further?

## 3. Not So Different Differences

Clearly, even from the extremely minimal description I have provided of “perspectivism” and what is often called “naturalism,” its Western counterpart, they are, in some respects extremely different. But given that the problem they raise for anthropological description is precisely about difference and its (potentially different) status in these cosmologies, it makes sense to be specific about what kind of difference this is.

To return to the distinction between referents of “nature” I made above, it is evident that “nature,” in so far as it refers to a set of objects, is indeed distributed differently across these two schemas. Within naturalist cosmologies, as they are represented, there is one world, and a “physical continuity” across bodies. “Nature,” in fact, in this sense, is what is responsible not for difference but for resemblance. It is the ground upon which comparative projects, cultural translation, and indeed all forms of communication take place. As an object, in other words, it plays exactly the mirror image role that “culture” occupies in perspectivist cosmologies. Take the following excerpt from Viveiros de Castro’s landmark 1998 article on the subject, for example:

We must remember, above all, that if there is a virtually universal Amerindian notion, it is that of an original state of undifferentiation between humans and animals . . . The original common condition of both humans and animals is not animality but rather humanity. (Viveiros de Castro 1998, 471; see also 2012, 31-32; Vilaça 2014, 323)

In other words, Amerindian cosmologies also include the notion of “an original state of undifferentiation between humans and animals.” Theirs is of humanity, ours of animality. Theirs of culture, ours of nature. That difference is of course enormously ethnographically significant. But it is worth noting that it is a perfect mirror-image inversion of what “nature” does for “us,” as Viveiros de Castro (1998, 470) himself points out: this is important not because it is a happy coincidence, but because, seen from the perspective of our anthropological meta-problem of what difference and resemblance mean, *the only thing that has changed is the object.* Both cosmologies possess notions of an originary, grounding resemblance between subjects. Both kinds of resemblance are given, essential features of life, not constructed or instituted by humans. In fact, if we were to reserve the word “natural” to mean “essential,” rather than allowing it also to refer to animals or the environment, then there would be no difference between the two cosmologies in this respect at all. I repeat that I am not, in any sense, discounting the importance of the perspective from which they do appear different: I am merely pointing out that from another they appear remarkably alike. And since likeness and difference are at issue here, that other perspective is worth noting.

Something similar is visible if we examine the opposite pole of the two cosmologies. “Culture” is what differentiates, in the schema of naturalism. It is what humans have, but we all have it differently. It is the object or medium of those projects of comparison, translation, communication, or conversion that take place against the background of natural uniformity. It is variable, constructed, and shifting. It is also, in those respects, the point for point opposite of “nature” as it is described in Amerindian cosmologies, though of course the objects to which the word “cultural” might attach remain the same. Take for example this description of the importance of bodily metamorphosis in perspectivism:

We need not be surprised by a way of thinking that posits bodies as the great differentiators yet at the same time states their transformability. Our cosmology supposes a singular distinctiveness of minds, but not even for this reason does it declare communication (albeit solipsism is a constant problem) to be impossible. . .Bodily metamorphosis is the Amerindian counterpart to the European theme of spiritual conversion. (Viveiros de Castro 1998, 481, 2014, 72; see also Vilaça 2005, 2011, 246-47)

Note again the equivalence established between the two schemas: (bodily) metamorphosis is (cultural) translation. That is, the objects transformed are entirely different in either case (souls and bodies): *but the fact of transformation is not*. Differences, in both schemas, are matters of contingent variability, not essential and stable properties. “We need not be surprised” at the notion of bodies as differentiators because they function as differentiators in precisely the same way as souls do for us.

## 4. The Nature of Difference

The reading of this material I have proposed thus far is not an alternative to the traditional one of perspectivist literature, in the sense that it is all present in the original explanations, but it does take a different perspective, one created by the equivocation over referents of “nature” and “culture.” One might tabulate these perspectives in the following manner, making use of some of the categories Viveiros de Castro himself suggests need redistributing when dealing with perspectivist cosmologies (Viveiros de Castro 1998, 469-70, 2014, 56).

Here is a fairly uncontroversial rendering of naturalism:

**Table:** 

**Nature**	**Physical objects**	**Resemblance**	**Essential**
Culture	Social objects	Difference	Instituted

Here is the reading of the perspectivist scheme that gives rise to worries about essentialism and the “ontologising” of difference:

**Table:** 

**Nature**	**Physical objects**	**Difference**	**Essential**
Culture	Social objects	Resemblance	Instituted

In this first reading of the perspectivist schema, the attributes of “instituted” and “essential” have remained attached to their relationship with culture and nature, while resemblance and difference have shifted. This reading thus sees particular differences, because they are now labeled “natural” as essential; as essentialist, in other words.

Here, instead, is the one I have been outlining:

**Table:** 

**Nature**	**Physical objects**	**Difference**	**Instituted**
Culture	Social objects	Resemblance	Essential

This second reading leaves the universal and the particular in the same relationship to the given and the instituted as in the naturalism schema. But this is more than simply a question of whether or not multinaturalism or “ontological” anthropology entails essentialism or not. It relates to the meta-problem of how the two schemas relate. Discard the middle table and we are left with the following:

Naturalism:

**Table:** 

**Nature**	**Physical objects**	**Resemblance**	**Essential**
Culture	Social objects	Difference	Instituted

Perspectivism:

**Table:** 

**Nature**	**Physical objects**	**Difference**	**Instituted**
Culture	Social objects	Resemblance	Essential

Recall that the question I suggested this article would attempt to address was that of how to articulate the relationship between cosmologies that are not merely different but differ over difference itself; contexts that are “radically” or “ontologically” different from our own. The difference between perspectivism and naturalism has often been taken to exemplify this difference, and a glance at the tables above is enough to make clear why this is the case: the two boxes on the right-hand side have been switched around in moving from the first to the second.

That is one perspective. Another perspective is that it is *only* the two boxes on the left-hand side that have switched around. In other words, the objects which differ from one another in particular, instituted, and spontaneous ways in naturalism (souls, for example, or ideas, or worldviews—“social” things) are not the same objects which differ from one another in particular, instituted, and spontaneous ways in perspectivism (bodies, for example, or worlds—“physical” things), but the fact of their difference and particularity being a matter of variation and transformation (institution and spontaneity, not essence and necessity) does not itself differ across the schemas. It is the objects of difference that have changed, not the nature of difference itself. The difference is over natural objects, not nature as essence. The difference between particular cases remains instituted, performed, and variable.

In other words, from the point of view of *what it means to be different*, rather than *what it is that is different*, the two cosmologies do not, in fact, appear to be so different. Let us call the former the “nature of difference.” If we proceed from the point of view laid out here—one that I again emphasize is only one such point of view; seen from another the two are as different as can be—then difference has the same nature in the two cosmologies: difference is instituted, not given, just as resemblance is given, and not instituted. The Other of the Other may not be the same as the Other of the Same (Viveiros de Castro 2004b, 12), but the relation between the Other of the Other and the Other of the Same is the same.

If we return to the table above, from the perspective I take here the boxes marked “nature” and “culture” actually tell us relatively little when detached from those to which they are analogized. The splendor and complexity of perspectivist universes for anthropology is not in the abstractive acrobatics of neologisms like “multinaturalism,” but in their meaning: that nature can refer to certain kinds of objects and at the same time to essence and givenness (Viveiros de Castro 2014, 75). The reason why statements of the form ‘*Culture is the Subject’s nature*’ need italicizing is that the words “culture” and “nature” are doing both those different sorts of work at the same time: “Culture,” *not* meaning something that has been instituted or constructed but the possession of a viewpoint or a soul, is *not* the subject’s body, but the subject’s given essence.

If we thus detach the boxes containing the words “nature” and “culture” from the tables we are left with a shuffling around of objects, such as bodies and souls. Nothing else has moved. “All the difference in the world,” from one perspective; not so different at all, from another.

This perspective, from which these cosmologies appear not so different, is that with which this article is occupied: the nature of difference. We can now frame a sort of meta-comparison, a comparison of comparisons, of the form Viveiros de Castro undertakes in outlining the notion of equivocation. Rather than focus on the object of such comparisons, however, in line with the argument so far, I will focus on their form or conception.

In perspectivist cosmologies there are things which are universal and things which are particular. The relationship of likeness or resemblance implied by universality is given and necessary. It is not the case that things that are universal just happen to be so, nor that they happen to have been made to be so, but that they must be so, and are essentially so. The relationship of difference implied by particularity is instituted and spontaneous. It is not the case that things that are different must be different, nor that such differences are unchangeable, but that they are produced, and may be otherwise.

Exactly the same is true of naturalist cosmologies.

Both sides of our meta-comparison, therefore, take attributes that are universal and shared to be essential and necessary when comparing, and both sides take attributes that are particular and differ to be instituted and spontaneous when comparing.

For our meta-comparison to resemble its component parts, then, which it must do for there is nothing else to it, *that fact itself must be given and necessary because it is shared across them*. It is, within the terms of the meta-comparison, universal, and what is universal within the parts of the meta-comparison is essential. Therefore, what is particular to the two sides of the comparison must equally be instituted and spontaneous, *because it differs across them*, just as what differs within them is instituted and spontaneous. In other words, just as discontinuities within the two cosmologies are matters of performance or construction (bodies or souls) so must be discontinuities across the two cosmologies: the “tactics” of “tactical quintessentialism.” By this expression I take Viveiros de Castro to mean the strategy of emphasizing not likeness, but difference. What I add here is that such emphasized differences are *necessarily* strategic or tactical. Whereas, the nature of difference, the essence of difference—as not, itself, essential or necessary—is, necessarily and essentially, the same across the schemas.

To state this more clearly: it is true ethnographically, as we have seen, that within the two schemas the difference between particular cases is instituted and spontaneous and what is universal is given and necessary. Therefore, that fact is itself universal across the two cases, and as such, logically, must also be given and necessary.

**Table:** 

		Meta-comparative difference		Meta-comparative resemblance
R=	**Naturalism**	**R=natural objects**	**Nature =essential**	**R= essential**
	Perspectivism	R=cultural objects	Cultural =essential	R= essential
D=	**Naturalism**	**D=cultural objects**	**Cultural =instituted**	**D=instituted**
	Perspectivism	D=natural objects	Nature =instituted	D=instituted
		Meta-comparative difference = instituted		Meta-comparative resemblance = essential

“All the difference in the world,” seen from this perspective, is actually always only a certain kind of difference, one which is instituted and spontaneous; and because it is always that kind of difference, it is necessarily and essentially always that kind of difference. All the difference in the world is the same. From this perspective, then, this paradigmatic example of “radical alterity” actually tells us that in a certain sense we are all the same, and we are necessarily and essentially so.

## 5. All the Difference in the World

The irony is not new. Michael Herzfeld makes this claim quite explicitly in his entry on “Essentialism” in the *Encyclopaedia of Social and Cultural Anthropology*: “Knowing this contested history [of eugenics in biology] enables us to focus on the *necessarily contingent* character of all forms of essentialism” (Herzfeld 1996, 189 my italics). It is also present as the implied opposite of ideas such as “tactical quintessentialism”: if essentialism is tactical, or strategic, what else can anti-essentialism be but given and necessary? But it is particularly notable that it is visible in the kind of contemporary work in anthropology that is alleged to be most sensitive to difference; indeed, to have revolutionized our notion of difference itself. Perhaps this is a transformation of a perspectivist problem: not “when everything is human, the human becomes a wholly other thing” (Viveiros de Castro 2014, 63), but “when everything is constructed, there are no wholly other things” (or, “hybridity is everyone,” as Sahlins, 1999, 411, once put it).

Why should this present a problem, given that it seems to match the conceptions of difference we find at work not only among cosmologies in which cultural objects are those which differ but even among those in which it is natural objects which do so? Granted, it suggests that “radical alterity” is perhaps not as radical as it first appears, but this in itself is not a novel suggestion (see, for example, Carrithers et al. 2010; Graeber 2015; Heywood 2012; Laidlaw and Heywood 2013), and neither is it especially germane to the expositional concerns of this article. I have noted from its outset that my purpose is not to question the ethnographic basis of claims about perspectivism, nor to exhaustively depict it, or its cosmological opposite.

Indeed, it is not necessary to stretch the imagination to think of instances in which those we might habitually describe as “naturalist” in their thinking treat difference as given and necessary, rather than spontaneous: such is not only true (some of the time) of some of the arguments concerning eugenics and its complicated political history that Vigh & Sausdal and Herzfeld presumably have in mind, but also (some of the time) of animal scientists (some of the time) of geneticists (Egorova 2018), or (some of the time) of mathematicians (e.g., Brodwin 2002; Candea 2010, 2013 see also Bloch 2008 on the “transcendental social”; and also Robbins 2016).

An ethnographer of Amazonia, one assumes likewise, will not find it difficult to think of instances either in literature or in their own experience in which the same is true of perspectivists—for example, it is clearly not the case that all animals possess the attribute of personhood (e.g., Viveiros de Castro 1998, 471, 2014, 57), and some have noted that it can be context-dependent (e.g., Willerslev 2007). Viveiros de Castro himself notes, for example, that

Amazonian cosmologies . . . have rich, equivocating resemblances to the distinction between the worlds of essence and appearance and could thus seem to lend themselves to a Platonic reading (the sole interest of which, however, would be to show how this Indian Platonism is merely apparent). (2014, 92)

The irony is obviously intended, but as he goes on to point out:

Indians are not Deleuzians, for they can just as much be Kantians as Nietzscheans, Bergsonians as Wittgensteinians, and Merleau-Pontyeans, Marxists, Freudians, and above all, Lévi-Strausseans. I believe I have even heard them referred to as Habermasians, and in that case, anything is possible. (2014, 93)

It is not especially surprising that when these cosmologies appear at the level of anthropological debates over how we ought to conceptualize difference, they sometimes do so in as schematic a form as that in which they have appeared here. Such is often the manner of our expositional strategies: something is held stable in order that a particular difference may be exposed (or a similarity, in the case of a related set of maneuvers that Sahlins called, *avant la lettre*, “ontological cocktails”; Sahlins 1999, 407). In the case I have here described it is the nature of difference itself that has been held stable by established literature, as something that cannot be stabilized, while other differences of “radical alterity” have been revealed.

There is, though, a cost to that maneuver, as there is to any similar one. The fact that these cosmologies only work in this way when abstracted, that both “perspectivists” and “naturalists” are neither all the time, remains true, even if not very useful to that perspective. So one would have to adopt another perspective in order to allow not only for “different natures” but also for different “natures of difference,” as I have been describing here.

Of course, re-thinking our assumptions about difference is precisely what anthropology’s “ontological turn” proposed to accomplish. That it has done so in terms of the kinds of objects which can differ is indisputable, but I have argued here that its conception of the nature of difference itself remains the one anthropologists have habitually preferred.

## 6. Styles of Difference

My suggestion, instead, is that we abandon any one conception of the nature of difference altogether, and attend instead to what we might think of as “styles” of difference (a more mischievous suggestion would be “cultures” of difference).^2^ People “do” difference differently all the time. Sometimes they treat it as something instituted and spontaneous—as is the case, for example, much of the time in my first fieldwork site (Heywood 2015a; 2015b; 2015c; 2018a; 2018b)—and sometimes they treat it as essential and necessary—as in the uncountable examples in which anthropologists critique or deconstruct such essentialism (e.g., Herzfeld 1987; Macdonald 1993; McDonald 1989; see also Bashkow 2006; Sylvain 2014). In addition, of course, these two poles may not exhaust the possible styles of thinking about difference, as I describe below.

We do not need, I think, a new “turn” to recognize that this is the case. One might argue, in fact, that we need precisely the opposite of any such attempt to systematize anthropological approaches to difference. The fact of “ontological” anthropological arguments producing remarkably similar accounts despite their proclaimed intention of taking difference more seriously than anthropology has done hitherto has been remarked upon already (see, for example, Heywood 2012; Candea 2017; Carrithers et al. 2010; Scott 2013, 2014 and for a response Holbraad 2017). My claim here has been somewhat more specific, in that I have pointed to the fact that such arguments do not simply resemble one another in haphazard fashion, but systematically: “all the difference in the world” is the same kind, or nature, of difference. There are few equivalents of other concepts in the anthropological canon which we allow ourselves to “naturalise” in the same way, deciding on behalf of interlocutors that if ever they show signs of straightforward essentialism it must be “strategic,” or that conceptions of transcendence or fundamentalism must, in order to be taken seriously, be reconciled with our preferentially immanentist and anti-representationalist philosophical inclinations.

Taking different “styles” of difference seriously is not in any sense the same as what we might think of as methodological essentialism (Heyes 2000). An attention to styles of difference would aim to shy away from pronouncements of any kind on the nature of difference, be they essentialist or otherwise. That this may be hard, if not impossible, to achieve all the time is no more evidence that it is a worthless enterprise than the fact that apprehending and attending to difference of any kind is difficult is evidence that we should abandon that most basic anthropological enterprise. Nor is it the same as asking how we ought to “take seriously” essentialism, for it proceeds from the premise that nobody is “essentialist” or otherwise all of the time. Indeed, in a sense this article is itself an example of this point: it is possible to read perspectivism in its schematic form as radically other to naturalism, as much of the literature on it does; it is equally possible to read them as radically similar, as I have done here. They are transformations of one another, like structuralist cosmologies. Which reading one employs has expositional consequences for the kinds of difference which emerge.

For a comparative example of what a focus on “styles of difference” might look like, there is a long-standing and growing body of literature in both developmental and social psychology on the workings of essentialism in children, adults, and groups (e.g., Barton and Komatsu 1989; Gelman 2003; Haslam et al. 2000; Keil 1992; Medin and Ortony 1989), some of which draws on psychological anthropology (e.g., Haslam et al. 2000, 115), but which has yet to have any major impact on social anthropology (though see Humphrey and Laidlaw’s argument that rituals are perceived as if they are “natural kinds”; Humphrey and Laidlaw 1994, 151-54). None of this work, as far as I am aware, is methodologically essentialist or makes claims to metaphysical essentialism, and much of it is at pains to make clear that it makes no essentialist claims of its own (e.g., Gelman 2003, 7; Haslam et al. 2000, 125; and in anthropology Humphrey and Laidlaw 1994, 153). But what it is also at pains to do is to examine, rigorously and empirically, the kinds of essentialist ideas people hold, the sorts of objects people hold them about, and the ways in which such notions develop. Susan Gelman, for example, writing of essentialism in children, distinguishes between metaphysical essentialism (the belief that essential differences reside in the world) and representational essentialism (the notion that they exist in our perception, between essentialism that defines categories (sortal), essentialism that defines functions or surface properties (causal), and essentialism that bears no relation to perceptions (ideal); Gelman 2003, 8-11). Likewise, Gelman, Medin, Barton and Komatsu, Keil, and others have distinguished between “natural kind” objects such as living beings and “artifacts” (human creations) in showing that both children and adults (in Euro-American contexts) often want to essentialize the former in terms of innate properties and the latter in terms of function (see, for example, Barton and Komatsu 1989, 444-45). Thus, a chair will be thought to cease to be a chair when it ceases to be something one can sit on, while a tiger would cease to be a tiger if its DNA were somehow altered. Hence, we might think of “structural” versus “functional” styles of difference. While this developmental literature has been taken up to some extent by social psychologists (see Haslam et al. 2000, 114-16), the question of how cultural or social contexts can impact upon practices of essentialization, or styles of difference, has yet to be addressed in depth (Gelman 2003, 6).

This sort of argument, incidentally, also draws on a rich seam of philosophical literature on the use of proper nouns that contradicts many of our preferentially Wittgensteinian views on language: “rigid designators,” as they are termed by Saul Kripke, most obviously in the form of names, are intended to pick out the object to which they refer in any possible world, and regardless of the guise in which it appears (Kripke 1980; Putnam 1975). Then there are the extensive debates within the feminist movement over the past several decades over exactly what kind of difference gender is, which have involved rich and productive differentiations between forms of difference, such as Cressida Heyes’ (2003, 37) typologies of essentialism, critiques of constructivist essentialism such as that of Elizabeth Spelman (1988), or Luce Irigaray’s and Iris Marion Young’s different attempts at reformulating an anti-essentialist form of gender difference (Irigaray 1985; Marion Young 1994; see also debates in Fuss 1989; and survey and argument in Stone 2004). Finally, some recent work in anthropology, in particular Rupert Stasch’s (2009) descriptions of the centrality of otherness to Korowai kinship relations (see also Reed 2004; Strathern 1996; Yarrow et al. 2015) perhaps heralds a newfound interest in the “post-relational” dimension of difference (Venkatesan et al. 2012).

My own work traverses two contexts in which two very different understandings of difference are at work, though the contexts themselves are only a matter of kilometers apart. My doctoral fieldwork was spent with queer activists in the Italian city of Bologna, who were, unsurprisingly, very much concerned with the nature of difference. In their case, the style of difference at issue was very much like that set out in the schemata of perspectivism and naturalism, as I have described at length elsewhere (Heywood 2018a; Heywood 2018b): much of what they did was concerned to demonstrate the absolutely non-essential nature of difference. This was true in some fairly obvious ways such as in regard to dress (wearing clothes and make-up chosen to combine stereotypically masculine and feminine appearances) and sexual behavior (polyamory and a refusal of categorizations such as “gay” or “lesbian”) but also, more deeply, of their style of political action. Fluid networks, which were aimed at sustaining differences of opinion and approach, were preferred to organized and hierarchical groups, and—strikingly—taking a collective position on a given issue by penning a manifesto or attending a march *en masse* could sometimes prove very difficult because the act of taking such a position could be seen as an attempt to impose identity. The fact that it was, in a sense, this insistence on the absolutely non-essential nature of difference that differentiated them in a fairly fixed manner from other activists and from those outside the LGBT+ movement echoes the paradox of essentially non-essential difference that follows from anthropological arguments about perspectivism, as I noted above.

My more recent fieldwork has taken place in the village of Predappio, just a few miles south of Bologna, but a world away politically (Heywood 2019). Predappio is the village in which Benito Mussolini was born, and since his body was buried there in 1957 it has become one of the premier sites of neo-fascist tourism in Italy, receiving around 100,000 such visitors a year. Yet it also lies in the heart of the most traditionally socialist region of Italy (Mussolini himself was a socialist for much of his early life), and the municipality has elected a succession of left-wing mayors ever since the end of the Second World War. The village has recently come to international attention because of its controversial ambition to build the country’s first “Museum of Fascism” in a building which used to house the local fascist party headquarters; opponents of the project decry it as an attempt to attract yet more “black” tourism, while supporters argue that such tourism is the result of a lack of education about the evils of fascism, precisely the problem that a museum would address. In this situation, and indeed in a wider global context in which the far-right appears to be undergoing a resurgence, identifying what “counts” as “fascist” is matter of significant consequence. The difference between being and not being fascist is a difference that people in Predappio—and indeed elsewhere—rarely think of as a blurred one, and a great deal of work goes into arguing over what it is that makes someone or something fascist or not, and shoring up that boundary when it appears: are Predappio’s buildings “fascist” because they were built under the auspices of the regime or by architects who were card-carrying party members, even though those same architects may have harbored anti-fascist sympathies, and the buildings look much like the University Library in Cambridge? How large a neo-fascist clientele is necessary to make a given business a “fascist” one, or is it the presence of fascist souvenirs for sale that determines the matter? Or the owner’s known or speculated political leanings? These sorts of arguments are a regular feature of everyday life in Predappio, and people have a range of answers to these questions; but very often they do have answers. That is, though they have different ideas about what it is that makes the difference between being fascist and not being fascist, that difference, for many, most definitely exists. The question of whether it is an insuperable one is an equally vital issue, and is currently being played out in debates over Predappio’s museum of fascism project, for example: there are some for whom Predappio’s status as “the Chernobyl of history,” as its current mayor puts it, is unchangeable and essential; it will be forever defined as a political “toxic waste dump” (Wu Ming 2018) by the accident of being the site of Mussolini’s birth, and by its reception of neo-fascist tourists. Others see in the museum project the first and only chance the village has had to save itself from this fate.

In aiming to attend to and apprehend difference in the world, we are habitually conceptually ascetic in our assumptions. We aim to take little, if anything, for granted as given and necessary. So why except the nature of difference itself from this premise? Why should the nature of difference be what we hold stable (as forever unstable) even as we allow all else to differ? I do not pose the question rhetorically: it regards what we understand to be the proper aim of our discipline. Whatever one’s answer, it is a question of method, not of metaphysics, and not asking it at all risks missing that fact, and our choices about what we take to be radical or otherwise: “radical alterity,” in respect of the nature of difference, is “alter” in exactly the same way as common or garden alterity, and anybody so different that their conception of difference is as given and necessary can be told they are simply mistaken (Candea 2011; Laidlaw and Heywood 2013).

An attention, on the other hand, to styles of difference would treat no form of alterity as “radical,” for something only appears to be radical if it departs from a set of assumptions about the nature of things, if we have made a metaphysics out of a methodological choice. We do not refer to particular religions as “radical,” nor to kinship arrangements, nor to forms of exchange. Why should “alterity” and conceptions of difference be themselves any different? To do so assumes that “all the difference in the world” does not, in fact, make much of a difference after all.

## Footnotes

1 I do not use the term “naturalism” in all the precise and specific senses in which it appears in the work of Philippe Descola (2013), but merely as an alternative shorthand for the structural opposite of perspectivism. As I make clear in the paper, the relationship between these terms and the ethnographic objects they are supposed to denote does not concern me here. I do not use either as descriptive terms for anything that Amerindians or Euro-Americans do or think. I use them as anthropological objects—concepts that appear and act in anthropological arguments over difference.

2 Akin to Jonathan Mair’s (2012) notion of “styles” of belief, or Mary Douglas’s (1996) “thought styles.”

## References

[bibr1-0048393120917947] Barton Michelle E. Komatsu Lloyd K. 1989 “Defining Features of Natural Kinds and Artifacts.” Journal of Psycholinguistic Research 18 (5): 433-47.

[bibr2-0048393120917947] Bashkow Ira 2006 The Meaning of Whitemen: Race and Modernity in the Orokaiva Cultural World. Chicago: University of Chicago Press.

[bibr3-0048393120917947] Bloch Maurice 2008 “Why Religion Is Nothing Special But Is Central.” Philosophical Transactions of the Royal Society B: Biological Sciences 363 (1499): 2055-61. 10.1098/rstb.2008.0007 PMC2606700 18292062

[bibr4-0048393120917947] Brodwin Paul 2002 “Genetics, Identity, and the Anthropology of Essentialism.” Anthropological Quarterly 75 (2): 323-30.

[bibr5-0048393120917947] Candea Matei 2010 “‘I Fell in Love with Carlos the Meerkat’: Engagement and Detachment in Human-Animal Relations.” American Ethnologist 37 (2): 241-58.

[bibr6-0048393120917947] Candea Matei 2011 “Endo/Exo.” Common Knowledge 17 (1): 146-50.

[bibr7-0048393120917947] Candea Matei 2013 “Suspending Belief: Epoché in Animal Behavior Science: Suspending Belief.” American Anthropologist 115 (3): 423-36.

[bibr8-0048393120917947] Candea Matei 2017 “We Have Never Been Pluralist: On Lateral and Frontal Comparisons in the Ontological Turn.” In Comparative Metaphysics: Ontology after Anthropology, edited by Pierre Charbonnier Salmon Gildas Skafish Peter, 85-107. London: Rowman & Littlefield Publishers.

[bibr9-0048393120917947] Carrithers Michael Candea Matei Sykes Karen Holbraad Martin Venkatesan Soumhya 2010 “Ontology Is Just Another Word for Culture: Motion Tabled at the 2008 Meeting of the Group for Debates in Anthropological Theory, University of Manchester.” Critique of Anthropology 30 (2): 152-200.

[bibr10-0048393120917947] Descola Philippe. 1986 La nature domestique: Symbolisme et praxis dans l’écologie des Achuar. Paris: Les Editions de la MSH.

[bibr11-0048393120917947] Descola Philippe 2013 Beyond Nature and Culture. Chicago: University of Chicago Press.

[bibr12-0048393120917947] Douglas Mary 1996 Thought Styles: Critical Essays on Good Taste. London: Sage.

[bibr13-0048393120917947] Egorova Yulia 2018 ‘DNA, Reconciliation, and Social Empowerment.’ The British Journal of Sociology 69 (3): 545-551. 30289160 10.1111/1468-4446.12597

[bibr14-0048393120917947] Fuss Diana, ed. 1989 Essentially Speaking: Feminism, Nature, and Difference. New York: Routledge.

[bibr15-0048393120917947] Gelman Susan A. 2003 The Essential Child: Origins of Essentialism in Everyday Thought. Oxford: Oxford University Press.

[bibr16-0048393120917947] Graeber David 2015 “Radical Alterity Is Just another Way of Saying ‘reality’: A Reply to Eduardo Viveiros de Castro.” HAU: Journal of Ethnographic Theory 5 (2): 1-41.

[bibr17-0048393120917947] Harris Oliver J. T. Robb John 2012 Multiple Ontologies and the Problem of the Body in History. American Anthropologist 114 (4): 668-679.

[bibr18-0048393120917947] Haslam Nick Rothschild Louis Ernst Donald 2000 “Essentialist Beliefs about Social Categories.” British Journal of Social Psychology 39 (1): 113-27. 10.1348/014466600164363 10774531

[bibr19-0048393120917947] Henare Amiria J. M. Holbraad Martin Wastell Sari, eds. 2007 Thinking through Things: Theorising Artefacts Ethnographically. London: Routledge/Taylor & Francis Group.

[bibr20-0048393120917947] Herzfeld Michael 1987 Anthropology through the Looking-Glass: Critical Ethnography in the Margins of Europe. Cambridge: Cambridge University Press.

[bibr21-0048393120917947] Herzfeld Michael 1996 “Essentialism.” In Encyclopedia of Social and Cultural Anthropology, edited by Alan Barnard Spencer Jonathan, 188-190. New York: Taylor & Francis.

[bibr22-0048393120917947] Heyes Cressida 2000 Line Drawings: Defining Women Through Feminist Practice. Ithaca: Cornell University Press.

[bibr23-0048393120917947] Heywood Paolo 2012 ‘Anthropology and What There Is: Reflections on “Ontology”’. The Cambridge Journal of Anthropology 30 (1): 143-151.

[bibr24-0048393120917947] Heywood Paolo 2015a. ‘Equivocal Locations: Being “Red” in “Red Bologna”’. Journal of the Royal Anthropological Institute 21 (4): 855-871.

[bibr25-0048393120917947] Heywood Paolo 2015b. ‘Freedom in the Code: the Anthropology of (Double) Morality’. Anthropological Theory 15 (2): 200-217.

[bibr26-0048393120917947] Heywood Paolo 2015c. ‘Agreeing to disagree: LGBTQ Activism and the Church in Italy.’ HAU: Journal of Ethnographic Theory 5 (2): 59-78.

[bibr27-0048393120917947] Heywood Paolo 2018a. After Difference: Queer Activism and Anthropological Theory. Oxford: Berghahn.

[bibr28-0048393120917947] Heywood Paolo 2018b. ‘Making Difference: Queer Activism and Anthropological Theory’. Current Anthropology 59 (3): 314-331.

[bibr29-0048393120917947] Heywood Paolo 2019 Fascism, Uncensored: Legalism and Neo-Fascist Pilgrimage in Predappio, Italy. Terrain 72: 86-103.

[bibr30-0048393120917947] Holbraad Martin 2012 Truth in Motion: The Recursive Anthropology of Cuban Divination. Chicago: The University of Chicago Press.

[bibr31-0048393120917947] Holbraad Martin 2017 “The Contingency of Concepts: Transcendental Deduction and Ethnographic Expression in Anthropological Thinking.” In Comparative Metaphysics: Ontology after Anthropology, edited by Charbonnier Pierre Salmon Gildas Skafish Peter, 133-59. London: Rowman & Littlefield Publishers.

[bibr32-0048393120917947] Humphrey Caroline Laidlaw James 1994 The Archetypal Actions of Ritual: a Theory of Ritual Illustrated by the Jain Rite of Worship. Oxford: Clarendon Press.

[bibr33-0048393120917947] Irigaray Luce 1985 This Sex Which is not One. Ithaca: Cornell University Press.

[bibr34-0048393120917947] Keil Frank C. 1992 Concepts, Kinds, and Cognitive Development. Cambridge: MIT Press.

[bibr35-0048393120917947] Kripke Saul 1980 Naming and Necessity. Harvard: Harvard University Press.

[bibr36-0048393120917947] Laidlaw James Heywood Paolo 2013 ‘One More Turn and You’re There’. Anthropology of This Century 7. http://aotcpress.com/articles/turn/.

[bibr37-0048393120917947] Latour Bruno 2009 “‘Perspectivism’: ‘Type’ or ‘Bomb’?” Anthropology Today 25 (2): 1-2.

[bibr38-0048393120917947] Macdonald Sharon. 1993 Inside European Identities: Ethnography in Western Europe. Oxford: Berg.

[bibr39-0048393120917947] Mair Jonathan 2012 “Cultures of Belief.” Anthropological Theory 12 (4): 448-66.

[bibr40-0048393120917947] Marion Young Iris 1994 ‘Gender as Seriality: Thinking about Women as a Social Collective’. Signs 19 (3): 713-738.

[bibr41-0048393120917947] McDonald Maryon 1989 “We Are Not French!”: Language, Culture, and Identity in Brittany. New York: Routledge.

[bibr42-0048393120917947] Medin Doug Ortony Andrew 1989 “Psychological Essentialism.” In Similarity and Analogical Reasoning, edited by Vosniadou Stella Ortony Andrew, 179-96. Cambridge: Cambridge University Press.

[bibr43-0048393120917947] Pedersen Morten Axel Empson Rebecca Humphrey Caroline 2007 “Editorial Introduction: Inner Asian Perspectivisms.” Inner Asia 9 (2): 141–52.

[bibr44-0048393120917947] Putnam Hilary 1975 ‘The Meaning of “Meaning”’. Minnesota Studies in the Philosophy of Science 7: 131-193.

[bibr45-0048393120917947] Reed Adam 2004 Papua New Guinea’s Last Place: Experiences of Constraint in a Postcolonial Prison. Oxford: Berghahn.

[bibr46-0048393120917947] Robbins Joel 2016 “What Is the Matter with Transcendence? On the Place of Religion in the New Anthropology of Ethics★.” Journal of the Royal Anthropological Institute 22 (4): 767-81.

[bibr47-0048393120917947] Scott Michael W. 2013 “The Anthropology of Ontology (Religious Science?).” Journal of the Royal Anthropological Institute 19 (4): 859-72.

[bibr48-0048393120917947] Scott Michael W. 2014 “To Be a Wonder: Anthropology, Cosmology, and Alterity.” In Framing Cosmologies: The Anthropology of Worlds, edited by Abramson Allen Holbraad Martin, 31-54. Manchester: Manchester University Press.

[bibr49-0048393120917947] Skafish Peter 2014 “Introduction.” In Cannibal Metaphysics: For a Post-Structural Anthropology, edited by de Castro Eduardo Batalha Viveiros Skafish Peter, 9-39. University of Minnesota Press.

[bibr50-0048393120917947] Spelman Elizabeth 1988 Inessential Women. Boston: Beacon Press.

[bibr51-0048393120917947] Stasch Rupert 2009 Society of Others: Kinship and Mourning in a West Papuan Place. Berkeley: University of California Press.

[bibr52-0048393120917947] Stone Alison 2004 ‘Essentialism and Anti-Essentialism in Feminist Philosophy’. Journal of Moral Philosophy 1 (2): 135-153.

[bibr53-0048393120917947] Strathern M. 1987 ‘An Awkward Relationship: the Case of Feminism and Anthropology’. Signs 12 (2): 276-292.

[bibr54-0048393120917947] Strathern M. 1996 ‘Cutting the Network’. Journal of the Royal Anthropological Institute 2 (3): 517-535.

[bibr55-0048393120917947] Strathern M. 2011 “Binary License.” Common Knowledge 17 (1): 87-103.

[bibr56-0048393120917947] Sylvain Renée 2014 “Essentialism and the Indigenous Politics of Recognition in Southern Africa: Essentialism and Recognition in South Africa.” American Anthropologist 116 (2): 251-64.

[bibr57-0048393120917947] Venkatesan Soumhya Candea M. Brun-Jensen C. Pedersen M. Leach J. Evans G. 2012 ‘The Task of Anthropology is to Invent Relations.’ Critique of Anthropology 32 (1): 43-86.

[bibr58-0048393120917947] Vigh Henrik Erdman Sausdal David Brehm 2014 “From Essence Back to Existence: Anthropology beyond the Ontological Turn.” Anthropological Theory 14 (1): 49-73.

[bibr59-0048393120917947] Vilaça Aparecida 2005 “Chronically Unstable Bodies: Reflections on Amazonian Corporalities.” The Journal of the Royal Anthropological Institute 11 (3): 445-64.

[bibr60-0048393120917947] Vilaça Aparecida 2011 “Dividuality in Amazonia: God, the Devil, and the Constitution of Personhood in Wari’ Christianity.” Journal of the Royal Anthropological Institute 17 (2): 243-62.

[bibr61-0048393120917947] Vilaça Aparecida 2014 “Culture and Self: The Different ‘Gifts’ Amerindians Receive from Catholics and Evangelicals.” Current Anthropology 55 (S10): S322–32.

[bibr62-0048393120917947] Viveiros de Castro Eduardo 1996 “Images of Nature and Society in Amazonian Ethnology.” Annual Review of Anthropology 25 (1): 179-200.

[bibr63-0048393120917947] Viveiros de Castro Eduardo 1998 “Cosmological Deixis and Amerindian Perspectivism.” The Journal of the Royal Anthropological Institute 4 (3): 469-88.

[bibr64-0048393120917947] Viveiros de Castro Eduardo 2004a. “Exchanging Perspectives: The Transformation of Objects into Subjects in Amerindian Ontologies.” Common Knowledge 10 (3): 463-84.

[bibr65-0048393120917947] Viveiros de Castro Eduardo 2004b. “Perspectival Anthropology and the Method of Controlled Equivocation.” Tipití: Journal of the Society for the Anthropology of Lowland South America 2 (1): 1-20.

[bibr66-0048393120917947] Viveiros de Castro Eduardo 2007 “The Crystal Forest: Notes on the Ontology of Amazonian Spirits.” Inner Asia 9 (2): 153-72.

[bibr67-0048393120917947] Viveiros de Castro Eduardo 2011a. “Zeno and the Art of Anthropology: Of Lies, Beliefs, Paradoxes, and Other Truths.” Common Knowledge 17 (1): 128-45.

[bibr68-0048393120917947] Viveiros de Castro Eduardo 2011b. “Zeno’s Wake.” Common Knowledge 17 (1): 163-65.

[bibr69-0048393120917947] Viveiros de Castro Eduardo 2012 “Immanence and Fear: Stranger-Events and Subjects in Amazonia.” HAU: Journal of Ethnographic Theory 2 (1): 27-43.

[bibr70-0048393120917947] Viveiros de Castro Eduardo 2014 Cannibal Metaphysics: For a Post-Structural Anthropology. Minneapolis: University of Minnesota Press.

[bibr71-0048393120917947] Wagner Roy 1981 The Invention of Culture. Revised and expanded edition A Phoenix Book. Chicago: University of Chicago Press.

[bibr72-0048393120917947] Willerslev Rane 2007 Soul Hunters: Hunting, Animism, and Personhood among the Siberian Yukaghirs. Berkeley: University of California Press.

[bibr73-0048393120917947] Wu Ming. 2018 Predappio Toxic Waste Blues. https://www.wumingfoundation.com/giap/2017/10/predappio-toxic-waste-blues-1-di-3.

[bibr74-0048393120917947] Yarrow Thomas Candea Matei Trundle Catherine Cook Joanna, eds. 2015 Detachment: Essays on the Limits of Relational Thinking. Manchester: Manchester University Press.

